# Glycogen accumulation, central carbon metabolism, and aging of hematopoietic stem and progenitor cells

**DOI:** 10.1038/s41598-020-68396-2

**Published:** 2020-07-14

**Authors:** Laura Poisa-Beiro, Judith Thoma, Jonathan Landry, Sven Sauer, Akihisa Yamamoto, Volker Eckstein, Natalie Romanov, Simon Raffel, Georg F. Hoffmann, Peer Bork, Vladimir Benes, Anne-Claude Gavin, Motomu Tanaka, Anthony D. Ho

**Affiliations:** 1 Department of Medicine V, Heidelberg University, Im Neuenheimer Feld 410, 69120 Heidelberg, Germany; 2 Molecular Medicine Partnership Unit Heidelberg, EMBL and Heidelberg University, 69120 Heidelberg, Germany; 3 Physical Chemistry of Biosystems, Institute of Physical Chemistry, Heidelberg University, Im Neuenheimer Feld 253, 69120 Heidelberg, Germany; 4 Center for Integrative Medicine and Physics, Institute for Advanced Study, Kyoto University, Kyoto, 606-8501 Japan; 5 Genomics Core Facility, European Molecular Biology Laboratory (EMBL), Meyerhofstrasse 1, 69117 Heidelberg, Germany; 6 Division of Child Neurology and Metabolic Diseases, Centre for Child and Adolescent Medicine, University Hospital Heidelberg, Im Neuenheimer Feld 430, 69120 Heidelberg, Germany; 7 Structural and Computational Biology Unit, European Molecular Biology Laboratory (EMBL), Meyerhofstrasse 1, 69117 Heidelberg, Germany; 8 Department for Cell Physiology and Metabolism, Centre Medical Universitaire, University of Geneva, Rue Michel-Servet 1, 1211 Geneva 4, Switzerland; 9 Present Address: Max Planck Institute of Biophysics, Max-von-Laue Straße 3, 60438 Frankfurt am Main, Germany

**Keywords:** Ageing, Haematopoietic stem cells, Stem-cell research

## Abstract

Inspired by recent proteomic data demonstrating the upregulation of carbon and glycogen metabolism in aging human hematopoietic stem and progenitor cells (HPCs, CD34+ cells), this report addresses whether this is caused by elevated glycolysis of the HPCs on a per cell basis, or by a subpopulation that has become more glycolytic. The average glycogen content in individual CD34+ cells from older subjects (> 50 years) was 3.5 times higher and more heterogeneous compared to younger subjects (< 35 years). Representative glycolytic enzyme activities in HPCs confirmed a significant increase in glycolysis in older subjects. The HPCs from older subjects can be fractionated into three distinct subsets with high, intermediate, and low glucose uptake (GU) capacity, while the subset with a high GU capacity could scarcely be detected in younger subjects. Thus, we conclude that upregulated glycolysis in aging HPCs is caused by the expansion of a more glycolytic HPC subset. Since single-cell RNA analysis has also demonstrated that this subpopulation is linked to myeloid differentiation and increased proliferation, isolation and mechanistic characterization of this subpopulation can be utilized to elucidate specific targets for therapeutic interventions to restore the lineage balance of aging HPCs.

## Introduction

Glycogen accumulation upon aging has been reported in cells such as nerves, neurons, astrocytes, and muscle cells^1–5^. Glycogen is the storage polyglucosan (PG) and periodic acid–Schiff (PAS) reaction has been established as the method to detect glycogen and other polysaccharides^6^. Glycogen content is usually low in blood cells but high levels of glycogen are characteristically found in the leukemia cells of patients with acute lymphoblastic leukemia (ALL)^7,8^. Before immuno- and molecular diagnostics for classification of acute leukemias has become routine, PAS-staining constituted an essential histochemical method for the classification of acute leukemias. Glycogen accumulation in form of PAS positive granules was prominently found in the blasts of ALL and was reported to indicate prognostic significance^8^.

In neurons of mice and Drosophila, accumulation of glycogen leads to neuronal loss, locomotion defects, and reduced life span^2^. Cellular glycogen deposits have been described in pathologic conditions such as diabetes or aging^1–3,5^. Genetic ablation of glycogen synthase in neurons improves neurologic function with age and extends life-span^3^. Evidence in the literature has therefore indicated that glycogen accumulation in cells and tissues might contribute to physiological aging and constitute a key factor regulating age-related functional decline^5,9^.

Using an unbiased and comprehensive proteomic and transcriptomic approach, our group has recently analyzed the molecular features of aging of human hematopoietic stem and progenitor cells (HPCs) as a model system for aging of other somatic stem cells. Among significant alterations in abundance of specific proteins in many pathways including those involved in DNA-repair, in proliferation, and in cellular metabolisms, the most unique and novel finding is the elevation of sugar metabolic enzymes in HPCs upon aging^10^. This upregulation of enzymes of the upper pathway of glycolysis is similar to that observed in cancer cells and is known as the Warburg effect^11,12^. Elevated glycolysis upon aging is simultaneously coupled with a shift in lineage differentiation potential of older HPCs towards granulocytic and myeloid lineages at the expense of lymphoid differentiation^10^. Lineage skewing is associated with a decline in HPC functionality and in immune defense, resulting in an increased susceptibility to infections^13^. In addition to glycolytic enzymes, other proteins involved in glycogen metabolism, glycogen phosphorylases brain and liver form (PYGB, PYGL), and glycogen debranching enzyme (AGL), phosphoglucomutase 1 (PGM1), were also significantly more abundant in older HPCs^10^.

The question arises whether this increase in glycolytic enzyme levels could be caused by an increase of these enzymes on a per-cell basis of the aging HPCs, or by the expansion of one of the subpopulations that have become more glycolytic than the others. With this background, we have assessed the alterations in glycogen content in HPCs from young and old human subjects via a semi-quantitative PAS analysis. With this new evaluation method, we have found that the up-regulation of glycolytic enzymes is due to the relative expansion of one of the CD34+ subpopulations that have become more glycolytic upon aging than the others. In addition, we examined if the subpopulation of HPCs with high glycogen levels reflects the subset that shows upregulated glycolytic enzyme activities and glucose-uptake capacity. In conjunction with analysis of single-cell transcriptomics data, we have provided evidence that the upregulated glycolysis in older HPCs is linked to myeloid lineage skewing and elevated proliferative activity.

## Results

### Glycogen accumulation in CD34+ HPCs

**Figure 1:**
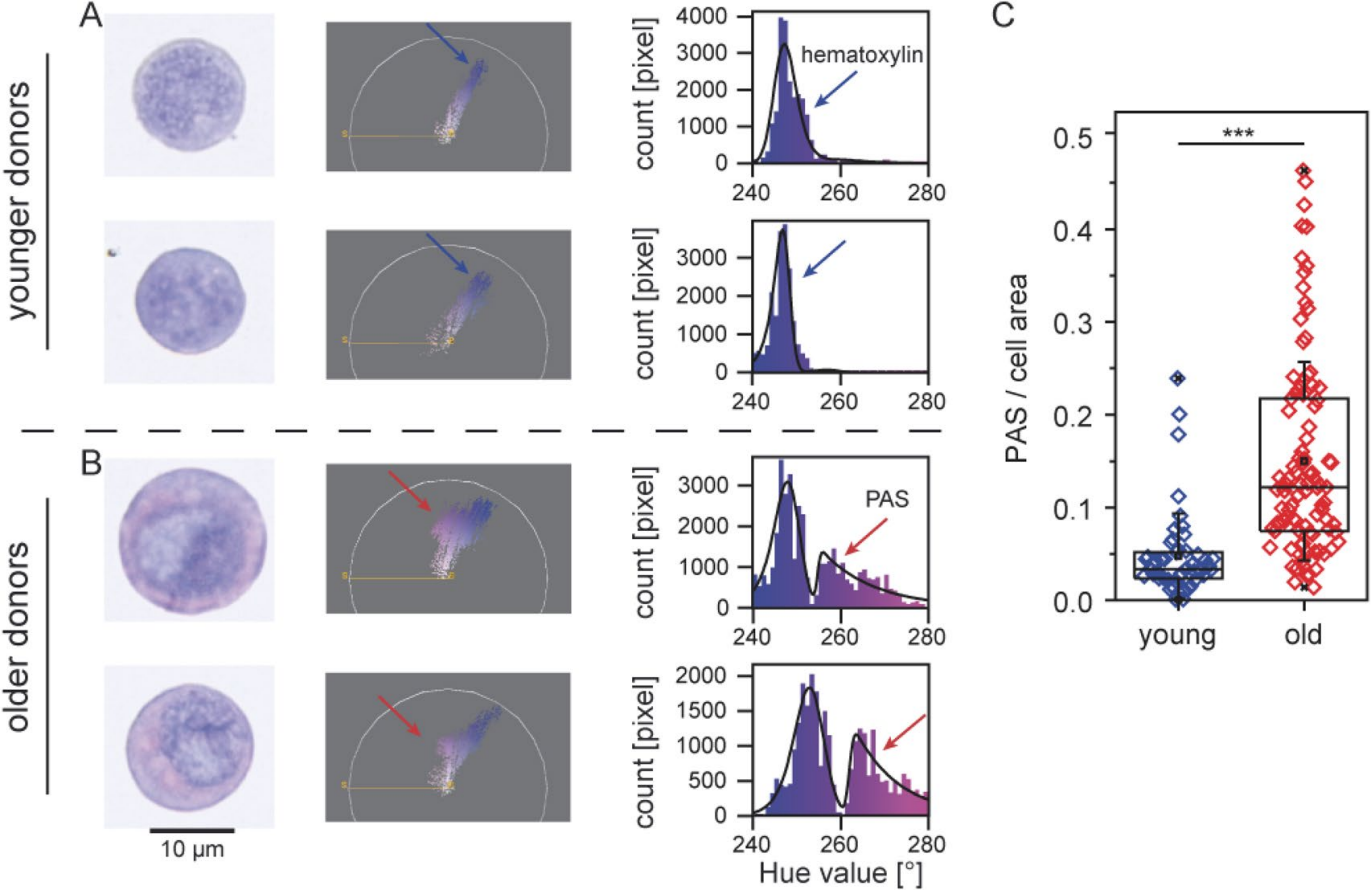
Semi-quantitative assessment of intensity of PAS reaction. (**A**) Example from a subject age < 35 years, (**B**) example from a subject > 50 years, arrows indicating signal from PAS and hematoxylin stained areas. (**C**) Results of the analysis in 6 human subjects, 3 younger (< 35 years) and 3 older (> 50 years). In subjects > 50 years, the average glycogen content was significantly higher than that found in human subjects < 35 years. The difference is statistically significant (one sided *t*-test, *p* = 1.2 × 10^–6^, N ≥ 15 CD34+ cells for each individual subject. Software: Fiji plug-in Color Inspector 3D.

Applying our newly developed semi-quantitative assessment of PAS positive (PAS+) content in individual cells as described under “Methods”, we have evaluated the glycogen content in the HPCs derived from younger (< 35 years; n = 3) versus older (> 50 years; n = 3) human subjects. Figure 1A depicts an example of the results from a young subject, and Fig. 1B an example of those from a subject > 50 years. At least 15 CD34+ cells were analyzed from every human subject, and the hue value of PAS+ content represents the glycogen content in each cell. In human subjects < 35 years, the glycogen content was relatively homogeneous. For older human subjects, the ratio between the signals from PAS stained regions and cell areas showed that the average glycogen content was 3.5 times higher and this difference was statistically highly significant (one-sided *t*-test, *p* = 1.2 × 10^–6^). The most prominent finding was, however, the heterogeneity of individual cells with high PAS+ content found in the older age group.

Thus, not only was the average glycogen content significantly higher in older subjects, but in addition, more heterogeneity was found among the older CD34+ cells. In approximately 25% of the cells, glycogen content was so high that it lied completely outside of the range found among young subjects, as depicted in Fig. 1C. The results of the analysis in 6 human subjects, 3 young and 3 older subjects are summarized in Fig. 1C. The difference between the two age groups is statistically significant (one-sided *t*-test, *p* = 1.2 × 10^–6^).

### Activities of representative glycolytic enzymes

Previous proteomics analysis showed a significant age-associated increase in abundance of proteins involved in the rate-limiting steps of the upper part of glycolysis, especially hexokinase 1 (HK 1), phosphofructokinase M, aldolase C (ALDO C) and triosephosphate isomerase 1 (TPI 1)^10^. In this study, we have assessed the enzyme activities of representative glycolytic enzymes HK, ALDO, TPI in the CD34+ cells derived from human subjects of different age groups. In addition, activity of adenylate kinase (ADK), an enzyme regulating glucose-driven energy metabolism was also studied. A major problem with enzyme assays for human samples is biological variance. To resolve this challenge, we have normalized enzymes that have been associated to a highly glycolytic phenotype to the ones associated with a normal glycolytic state, the latter being pyruvate kinase “high affinity”. As shown in Table S1 and in Table S2, this internal normalization was able to eliminate a lot of noise and accounts for individual differences of overall glycolysis activity.

**Figure 2:**
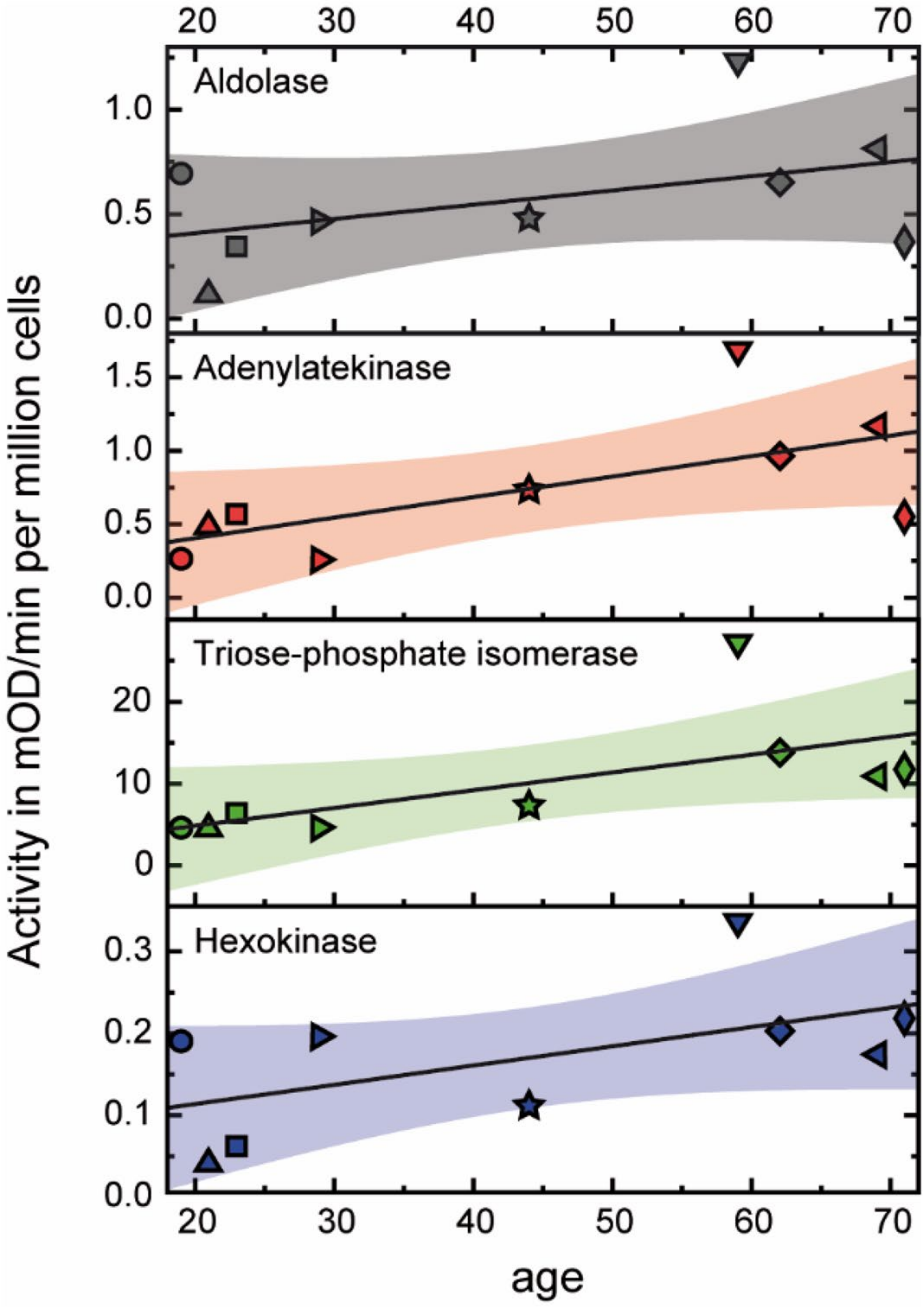
Ezymatic activities of glycolytic enzymes and adenylate kinase in CD34+ cells with aging. Enzymatic activities of aldolase (ALDO), adenylate kinase (ADK), triose-phosphate isomerase (TPI), and hexokinase (HK) plotted as a function of age. Scatter plot of enzyme activities, linear fits (solid lines), and 95% confidence band of fitting curves (colored areas) are presented. Individual symbols represent the corresponding enzyme activity in each of the 9 human subjects. The increase in enzyme activities is statistically significance for adenylate kinase (ADK; *p* = 0.02), for triose-phosphate isomerase (TPI; *p* = 0.01), and for hexokinase (HK, *p* = 0.04), but not for aldolase (ALDO; *p* = 0.07). Software: OriginPro 2018b.

The activities of these enzymes were assayed in nine individuals ranging from 19 to 71 years of age. The results are presented in Table 1 and Fig. 2. Analysis of z-standardized data revealed increasing activities of these enzymes (r^2^ of trend: ALDO, 0.21; ADK, 0.43; TPI, 0.42; HK, 0.33) with age. The activities of ADK, TPI and HK were significantly increased in the elderly cohort (59-, 62-, 69-, 71-years) compared to the young cohort (19-, 21-, 23-, 29-years), as summarized in Table 1. The statistical analysis is shown in Table 2. Thus the previous observation of increase in abundance of glycolytic enzymes in proteomics can be validated by measurements of enzyme activities.

**Table 1:** The activities of key enzymes in glucose-driven energy metabolism with age.

ID	Age	Gender	Activity normalized based on pyruvate kinase				Pyruvte kinase high affinity
			Aldolase	Adenylate kinase	Triose-phosphate isomerase	Hexo-kinase	
219	19	f	0.69	0.27	4.87	0.19	20.52
309	21	f	0.12	0.51	4.94	0.04	21.72
308	23	m	0.34	0.59	6.75	0.06	5.81
214	29	m	0.46	0.27	4.90	0.20	7.03
307	44	m	0.48	0.76	7.70	0.11	18.54
302	59	m	1.23	1.74	28.33	0.34	22.43
305	62	f	0.65	1.00	14.48	0.20	11.28
303	69	m	0.81	1.21	11.41	0.17	9.58
221	71	f	0.37	0.57	12.33	0.22	6.23

The enzymatic activities of aldolase, adenylate kinase, triosephosphate isomerase, and hexokinase in 9 human subjects were assessed (age range: 19–71 years). To overcome the significant biological variance, we have normalized the function of each enzyme by pyruvate kinase “high affinity”.

**Table 2:** Statistical analysis.

	Activity normalized based on pyruvate kinase				Pyruvte kinase high affinity
	Aldolase	Adenylate kinase	Triose-phosphate isomerase	Hexo-kinase	
One-sided *t*-test					
Mean	0.57	0.77	10.63	0.17	
SD	0.32	0.48	7.53	0.09	
Correlation coefficient					
Mean young (< 40)	0.41	0.41	5.36	0.12	13.77
Mean old (> 50)	0.76	1.13	16.64	0.23	12.38
* p* value	0.07	0.02	0.01	0.04	0.40

After z-standardization. one-sided *t*-test was used based on the hypothesis that activities were higher in older subjects than younger subjects. The data from subject 307 (44 years) in Table 1 were excluded from the analysis, as they represent the median values.

### Classification of HPCs subsets by glucose uptake capacity

**Figure 3:**
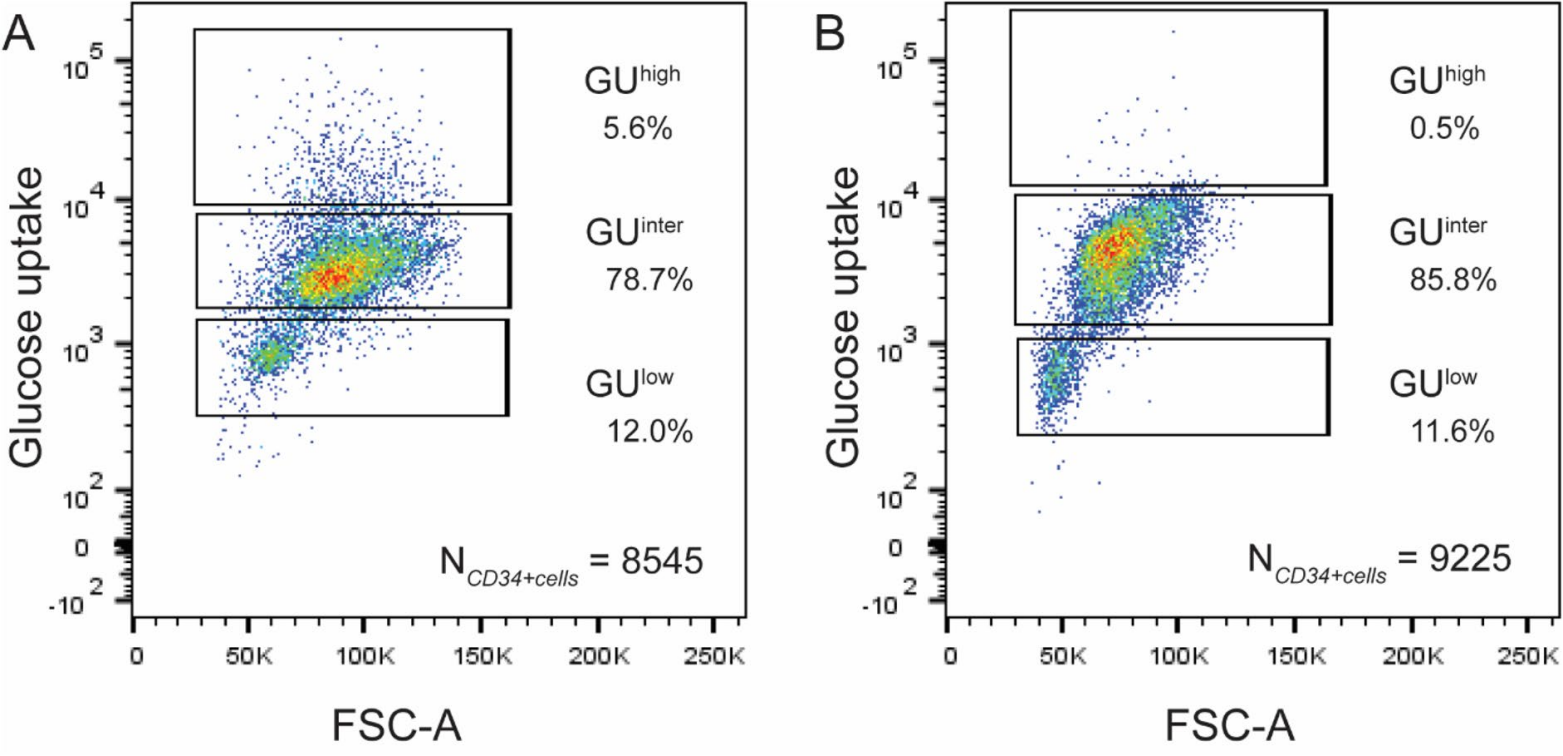
Glucose uptake capacity of the CD34+ HPCs. Glucose uptake capacity of the total CD34+ cells was assessed by the Cayman’s Glucose Uptake Assay Kit. Incubation for 30 min with 1.75 μg/ml 2-NBDG yielded a dose-dependent uptake of glucose into the CD34+ cells. The latter could then be separated according to their respective levels of glucose uptake by a FAC-Sorter into three distinct subpopulations according to the glucose uptake (GU) capabilities: GU^low^, GU^inter^, and GU^high^. (**A**) An example of separation of CD34+ cells from a human subject > 50 years according to GU capacity. (**B**) An example of separation of CD34+ cells from a young subject (< 35 years). Only GU^low^ and GU^inter^, but scarcely any GU^high^ cells could be detected. Software: FlowJo v10.6.2.

We subsequently determined the glucose uptake (GU) of the CD34+ cells. Preliminary experiments showed that incubation for 30 min with 75 µg/ml NBDG yielded a dose-dependent uptake of glucose into the CD34+ cells. As the cells could be separated according to their glucose uptake (GU) capacity by means of a FACS sorter, the CD34+ cells derived from human subjects older than 50 years could be consistently separated into three distinct subpopulations: CD34+ cells with low (GU^low^), intermediate (GU^inter^), and high (GU^high^) GU capacity (Fig. 3A). In human subjects younger than 35 years (Fig. 3B), most of the CD34+ cells were in the GU^inter^ and GU^low^ range and there were scarcely any CD34+ cells with GU^high^. Table 3 depicts a list of the fractions of GU^high^, GU^inter^, and GU^low^ cells from young (< 35 years, n = 3) versus older (> 50 years; n = 7) human subjects. W hereas there is no difference in the percentages of GU^inter^ and GU^low^ fractions between young and older subjects, the difference in GU^high^ is significant (*p* = 0.02, one-sided *t*-test).

**Table 3:** Fraction of GU^high^, GU^inter^, and GU^low^ cells from young (≤ 35 years) versus older (> 50 years) healthy human subjects.

Population	Fraction in young subjects [%] (n = 3)	Fraction in older subjects [%] (n = 7)	Note
GU^high^	1.7 ± 1.5	5.4 ± 3.5	The fraction of GU^high^ cells from older donors was significantly higher than that from younger donors (one-sided *t*-test, *p* = 0.02)
GU^inter^	66.5 ± 36.9	66.4 ± 22.5	No significant difference
GU^low^	31.8 ± 36.7	28.2 ± 21.7	No significant difference

**Figure 4:**
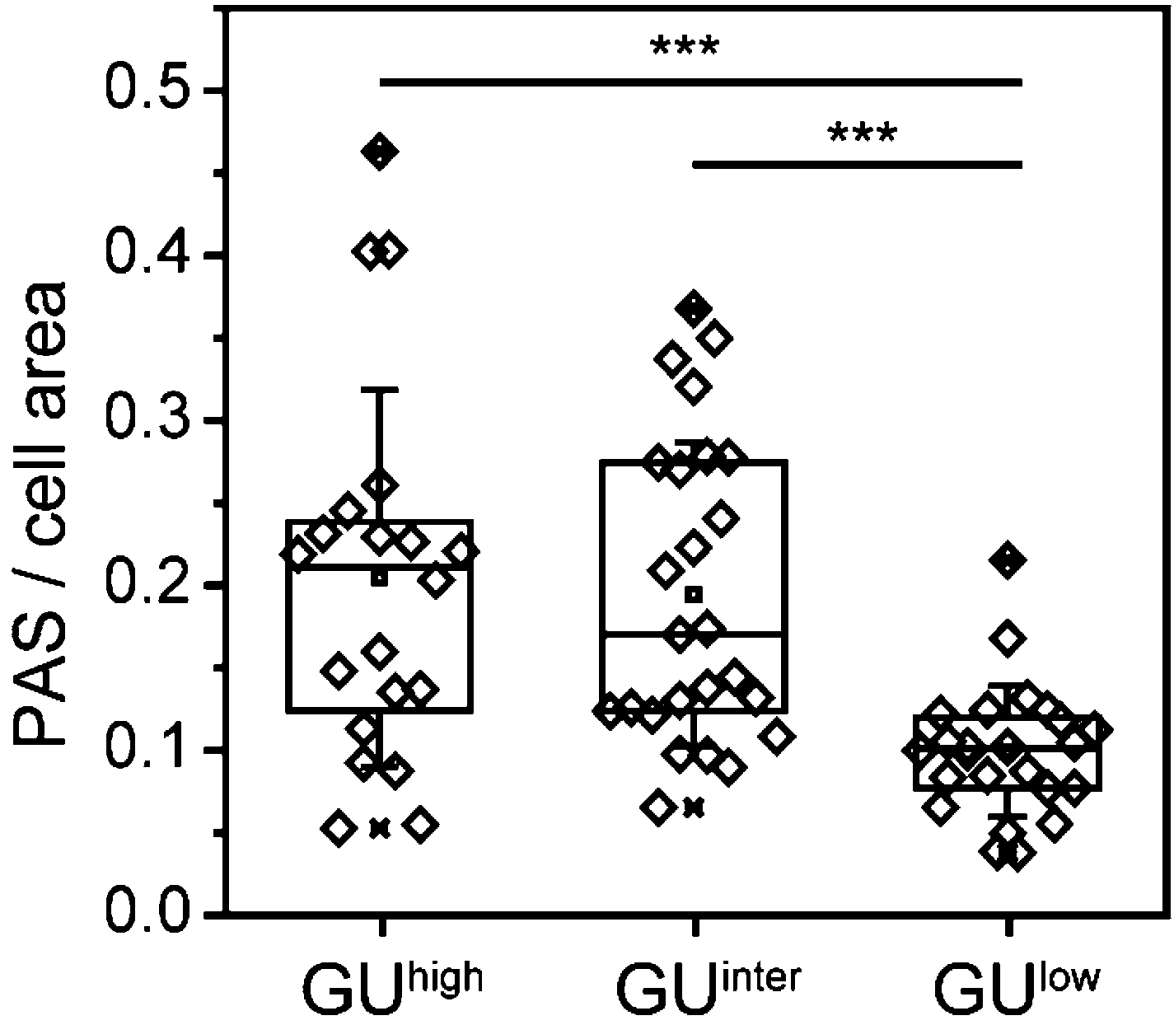
PAS signals in CD34+ cells from subjects > 50 years after separation according to glucose metabolism. Difference between ratios of PAS stained areas in GU^low^ population versus the population with high glucose uptake (GU^high^) is highly significant (one-sided *t*-test, *p* < 3.5 × 10^–4^, N ≥ 20 CD34+ cells for each population). Software: OriginPro 2018b.

Cytospins of these three fractions from the same individual human subjects were made in two of the elderly subjects and PAS reaction on these smears was performed. The results of these studies are summarized in Fig. 4. The PAS analysis demonstrated that the GU^low^ correlated precisely with CD34+ cells with low to undetectable ratios of PAS/cell areas, GU^inter^ with intermediate glycogen content (PAS), and GU^high^ with very high glycogen content.

### Single-cell RNA sequencing of CD34+ HPCs

To examine the relationship between an increase in abundance of the enzymes of the “preparatory” phase of the glycolytic pathway and the lineage skewing of HPCs towards myeloid differentiation upon aging, we previously analyzed the transcriptomes of 519 single-cell sorted CD34+ cells originating from young (*n* = 2) and old (*n* = 2) human subjects^10^. Based on the abundance levels of the mRNA markers of lymphoid or myeloid differentiation, we have categorized each individual HPC accordingly. The remarkable finding was that the age-dependent increase in glycolytic enzymes was found only in the myeloid-primed subset of CD34+ cells. The work flow of the analyses is depicted in Figure S3 and the number of cells per age group and the quality control data have been described in detail^10^.

On re-analyses of these transcriptomics data, we performed a Gene Ontology (GO) analysis and Pathway analysis. The list of genes that served as markers for myeloid versus lymphoid development is listed in Table S3. For the GO analysis the R packages clusterProfiler^14^ and topGO^15^ were applied; for the pathway analysis we conducted a Fisher Exact test on pathways extracted from the Reactome Database^16^. The results of GO and Pathway analyses are summarized in Supplementary Document. Regulation was defined according to the upward or downward slope between young and old subjects.

**Figure 5:**
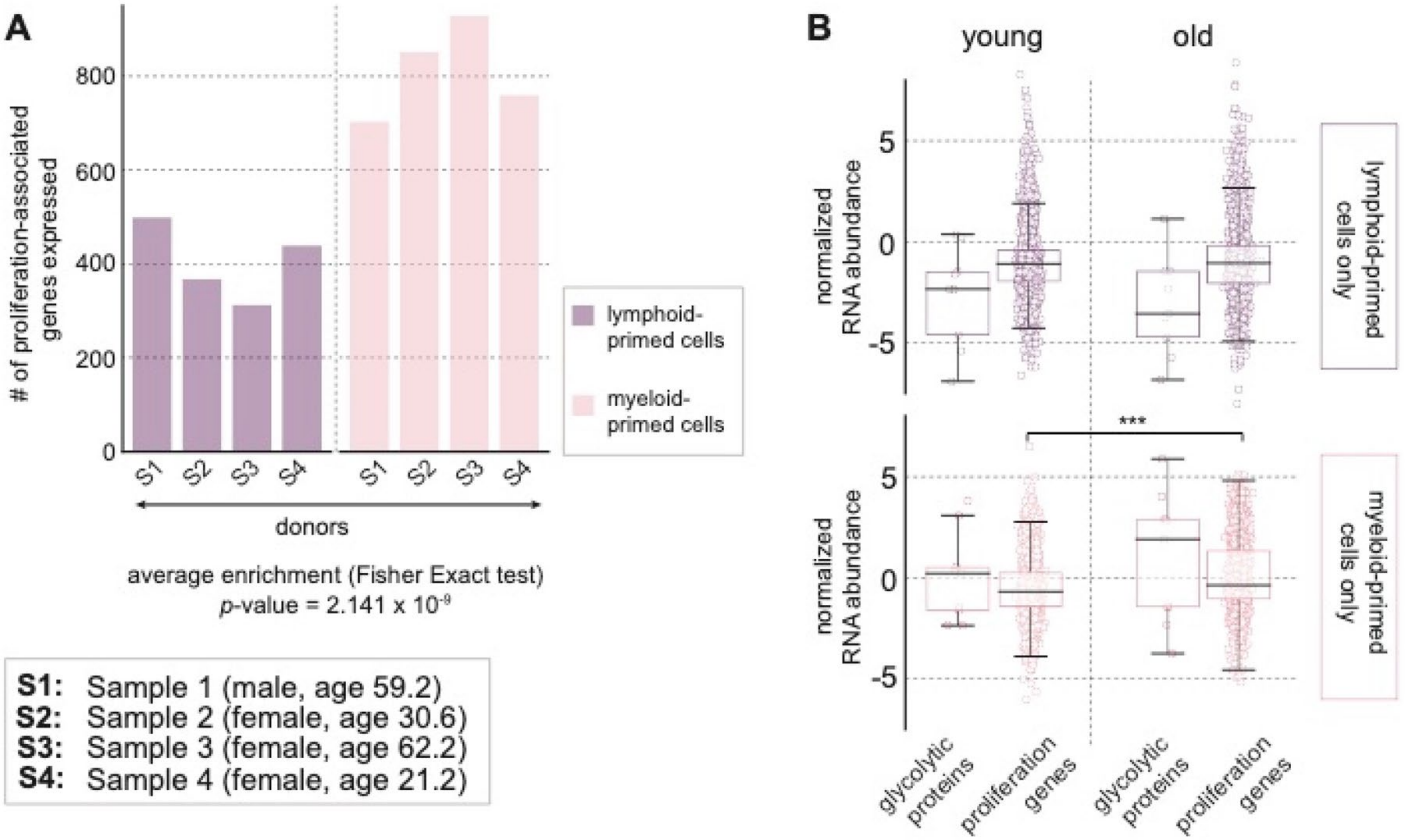
Influence of aging detected by single-cell RNA sequencing. (**A**) Proliferation-associated genes were significantly increased in the myeloid primed cells. The bar-plots show the number of proliferation-associated genes as defined by ontology in lymphoid (purple) versus myeloid-primed (pink) CD34+ cells across the 4 donors. The RNA markers for myeloid versus lymphoid differentiation potential are listed in Table S1. The average enrichment was calculated using *Fisher Exact test* and indicated an overall increase in proliferation-genes in the myeloid-primed subset of CD34+ cells. (**B**) Up-regulation of expression of proliferation-associated genes in older subjects. The first two boxplots in each row correspond to the gene expressions in lymphoid-primed CD34+ cells. and the second two box-plots show the corresponding gene expressions in myeloid-primed cells. The differences between young and old both in glycolytic and in proliferation-associated proteins among the lymphoid-primed cells were not significant. Among the myeloid-primed cells. parallel to the increase in glycolytic proteins. there was a significant increase in abundance of proliferation-associated genes in older human subjects. Software: Python 2.7. (Accession number of single cell RNA-sequencing data: https://www.ncbi.nlm.nih.gov/geo/query/acc.cgi?acc=GSE115353).

Figure 5 summarizes the results of these analyses and demonstrates that the genes associated with proliferation were increased in the myeloid primed cells. In Fig. 5A, the bar-plots show the number of proliferation-associated genes as defined by ontology in lymphoid (purple) versus myeloid-primed (pink) CD34+ cells across the 4 subjects. The average enrichment was calculated and indicated an overall increase in proliferation-genes in the myeloid-primed subset of CD34+ cells. Figure 5B demonstrates that the expression of proliferation-associated genes was upregulated in older subjects. The first two boxplots in each row correspond to the gene expressions in lymphoid-primed CD34+ cells, and the second two box-plots show the corresponding gene expressions in myeloid-primed cells. The distribution of mRNA abundance levels for glycolytic proteins among the lymphoid-primed CD34+ cells was lower in older subjects and the differences between young and old both in glycolytic as well as in proliferation-associated proteins among the lymphoid-primed cells were not significant. Whereas among the myeloid-primed cells, there was a significant increase in abundance of proliferation-associated genes in older human subjects, parallel to the increase in glycolytic proteins. Figure S4 shows the relationship between aging of CD34+ cells and co-expressions of CD38, CD90, CD45RA and CD49f obtained from the single-cell transcriptomics study.

## Discussion

Aging is associated with accumulation of glycogen granules in brain tissues, neurons^1–3^, muscles^4^, and in liver tissues^5^. In most studies, periodic acid–Schiff (PAS) reaction has been used as the standard method to detect glycogen and polysaccharides^6,7^. In bone marrow or blood cells, no relationship between accumulation of glycogen granules and aging has yet been described. For acute lymphoblastic leukemia, the degree of PAS reactivity in bone marrow cells was reported to indicate clinical prognosis^8^, but was not confirmed by other authors^17^.

Since the PAS staining method was established in 1946, it has remained the most widely used qualitative assay for carbohydrates and glycogen. Interest in assessment of glycogen granules and their relationship to aging has led to recent adaptations of the PAS reaction as a diagnostic tool and for quantitatively colorimetric analysis^5,18,19^. We have assessed the relationship between glycogen accumulation and aging at a single cell level by a new approach. To semi-quantitatively assess the PAS-positive content found in individual cells, specifically HPCs, our computer assisted evaluation system was able to estimate and compare semi-quantitatively the glycogen content of CD34+ cells from young (< 35 years) versus older (> 50 years) subjects in a reproducible manner. The average ratio between the signals from PAS stained regions and the cell area was 3.5 times higher for older subjects. More remarkably, we found a significantly higher heterogeneity in glycogen content among the HPCs in the older age group.

Previous comprehensive proteomic studies from our group demonstrated that aging HPCs are uniquely characterized by upregulated carbon metabolism^10^. The proteome data also revealed many other age dependent alterations in HPCs that were described fragmentarily by other authors using genomic and transcriptomic studies on HPCs. Our unique finding of upregulated glycolysis and anabolic metabolism upon aging of human HPCs was novel and remarkable. The question arises whether this is caused by elevated glycolysis and metabolism of the whole HPC population on a per cell basis, or by a subpopulation that has evolved upon aging.

Using our new approach, we have demonstrated that the elevated glycolysis in older HPCs is caused by the expansion of one HPC subset that has become more glycolytic than the others and is not based on increased glycolysis in the total HPC population. Single cell transcriptomics data have provided evidence that the HPC subset that is more glycolytic is exclusively linked to myeloid differentiation, and to enhanced metabolic as well as proliferative activities. All these results indicate that this subpopulation of CD34+ cells, identified by high glycogen content and PAS reactivity, represents a cell clone that might play a major role in the aging process of hematopoiesis.

Compatible with our results and using single-cell transcriptomics, Kirschner et al. identified a distinct subpopulation of old, myeloid biased, HPCs carrying a p53 signature indicative of stem cell decline alongside pro-proliferative JAK/STAT signaling. By challenging HPCs with an active form of JAK2 (V617F), they showed an expansion of the p53-positive subpopulation in old mice^20^. Their results suggested a cellular heterogeneity in the onset of HPC aging, with the responsible subpopulation characterized by myeloid lineage skewing and elevated proliferation.

To validate the alterations in abundance of glycolytic proteins, we have performed functional assays of representative glycolytic enzymes as well as ADK in the CD34+ cells derived from 9 different human subjects with ages between 19 and 71 years (Table 1 and Fig. 2). These enzymatic assessments have provided evidence for, and validated nicely, the significant increase in abundance of glycolytic enzymes in CD34+ cells derived from older subjects.

For further in-depth mechanistic studies of this specific subset responsible for the aging process of HPCs, isolation of these CD34+ cells according to glycolytic and metabolic characteristics represents a pre-requisite. Provided with the knowledge gained from the semi-quantitative PAS reactivity, and applying glucose uptake assay, we have demonstrated that the variations in glycolytic metabolism can now be exploited to separate and isolate the HPCs according to their GU capacity. The advantage of this method is that vital and intact HPCs can be harvested for further functional studies. From the bone marrow of older human subjects, we were consistently able to separate the HPCs in GU^high^, GU^inter^ and GU^low^ subpopulations. For subjects younger than 35 years, the GU^high^ subset was, however, hardly detectable.

Single cell RNA sequencing analysis has provided further evidence that the re-wiring of central carbon metabolism was found in the myeloid-primed subpopulation of HPCs and not in the lymphoid-primed cells, nor in the CD34+ subpopulation that showed no lineage commitment^10^. Re-analyses of these transcriptomic data in the context of the present study have demonstrated that proliferation-associated gene expressions are up-regulated in myeloid primed cells from elderly donors but not in lymphoid-primed CD34+ cells. The results of our re-analysis of single-cell transcriptomics data also support the observation reported by Kirschner et al. They demonstrated that a subpopulation with myeloid bias and elevated proliferation drives aging-related functional decline in murine HPCs^20^.

In the past years, large scale genomic studies of human leukocytes have indicated that clonal hematopoiesis of indeterminate potential (CHIP) is a frequent phenomenon found in elderly human subjects^21–24^. CHIP is characterized by the presence of an expanded somatic blood-cell clone without clinically overt hematologic abnormalities. The incidence increases with age and is associated with an increased risk of hematologic cancer and especially a doubling in the risk of developing atherosclerotic cardiovascular disease and mortality^21,22^. Large cohort studies have further demonstrated that CHIP is associated with an expansion of HPCs carrying recurrent somatic mutations which are common in myelodysplastic syndromes (MDS) and acute myeloid leukemia (AML)^24,25^. These CHIP-associated mutations provide a selective advantage to the HPCs in which they occur. Such clones are coupled with myeloid lineage skewing at the expense of lymphoid development^25–27^.

Given our results in the present study and in conjunction with our proteomics data, the subpopulation with elevated glycolysis and high PAS reactivity in elderly human subjects is closely associated with myeloid lineage skewing, increased proliferative activity, and is probably responsible for the aging process of hematopoiesis. We speculate that the elevated glycolysis in this subset, coupled with myeloid lineage skewing and elevated proliferation, probably plays a major role in the aging process. The elevated central carbon metabolism, linked with myeloid bias and increased proliferation might reflect an increased demand for production of cofactors and intermediates for nucleotides, lipids and amino acids synthesis for proliferation^12^. In conjunction with our present knowledge on emergence of CHIP upon aging, the subpopulation that have become more glycolytic might represent the subset of HPCs associated with CHIP and the corresponding somatic mutations.

The ability to isolate the HPCs according to their glucose metabolic characteristics represents a prerequisite for in-depth studies on the functional and mechanistic differences, and to determine whether recurrent somatic mutations responsible for CHIP could be identified and enriched in this subpopulation. It is also expected that GU^high^ cells might have distinct properties with respect to other metabolites to meet the particular metabolic demands of these cells. Similar to the Warburg effect in cancer cells, these HPCs might have become overly dependent on the aberrant glycolytic pathways. This difference to normal development could be exploited therapeutically^12,28^. The metabolic signatures of these CD34+ cells might reveal specific targets for therapeutic interventions to restore the balance between myeloid and lymphoid differentiation. Isolation of relative pure subpopulations according to their metabolic characteristics represents a fundamental initial step.

## Methods

All methods were carried out in accordance with relevant guidelines and regulations.

### Isolation of hematopoietic stem and progenitor cells (HPCs)

Bone marrow samples were harvested from human subjects from the posterior iliac crest using a Yamshidi needle, with aspirations at 5–7 different levels of approximately 10 ml at each level^29^. The study has been approved by the Ethics Committee for Human Subjects at the University Heidelberg and written informed consent was obtained from each individual. We recruited 17 subjects with the age ranging from 21 to 71 years for the different experiments within this study. The bone marrow aspirates were processed by FICOLL density fractionation for isolation of mononuclear cells (MNCs). After staining with CD34-APC and CD14-PE (both from BD Biosciences, San Jose, CA) the CD34+ cell population was isolated using a FACSAria II flow cytometry cell sorter (BD Biosciences). After Fluorescence Activated Cell Sorting (FACS), the sorted cells were analyzed for their purity by reanalyzing an aliquot of the respective cell population^10^.

### Periodic acid–Schiff (PAS) staining

Cytopins were prepared, according to the manufacturer´s instructions, utilizing the Cytospin 2 centrifuge (Thermo-Shandon, Pittsburgh, PA, USA). Shortly, 10.000 CD34+ cells were resuspended in 100 µl PBS and pipetted inside of a special plastic chamber connected with a special metal clip to a glass slide and a filter card (Thermo-Shandon, Pittsburgh, PA, USA) and centrifuged at 700 rpm for 5 min.

The generated cytospins were stained using the Periodic acid Schiff (PAS) method (395B-1KT, Sigma-Aldrich, St Louis, MO), for detection of the glycogen storage in each cell, according to the manufacturer’s instructions. Shortly, cells were fixed with 4% paraformaldehyde, incubated with 0.5% periodic acid solution for 5 min, and stained with Schiff's reagent for 15 min. This is followed by counterstaining with hematoxylin solution for 1.5 min. All steps were performed at room temperature, and cells were rinsed with tap water after each step^30^.

### Semi-quantitative assessment of glycogen content

To semi-quantitatively assess the differences in glycogen content among the CD34+ cells and especially differences across life span, we developed a new image analysis platform. Bright-field images of smear samples of HPCs stained with PAS were taken with an Olympus BH-2 microscope, equipped with a Panasonic GP-KH232HM CMOS camera (8-bit RGB) and a 100× oil immersion objective. All images were taken with the identical setting of illumination using the same camera to gain a stable, constant background profile. It should be noted that the images were taken within 4 months after smear samples had been prepared, as samples showed significant degradation of color after 6 months of staining. The obtained images were analyzed by using the ImageJ/Fiji software^31^. To compensate inhomogeneous background illumination and sensitivity of the sensor array, we corrected the background according to $I_{c} = 255 \times \left( {I_{s} - I_{d} } \right)/\left( {I_{b} - I_{d} } \right)$. $I_{c}$ stands for the image after correction, while $I_{s}$ for the raw image of specimen. $I_{b}$ and $I_{d}$ are the background signals acquired with open and blocked light paths, respectively^32^.

To evaluate the PAS signals from the obtained images, we first subjected the background-corrected images to the commonly used color deconvolution with defined color vectors^33^. Unfortunately, slight differences in the tones made the color deconvolution practically impossible (Figure S1 in Supplementary Material). To overcome the difference in tones, we converted the background-corrected images from RGB (red–green–blue) to HSB (hue-saturation-brightness) representation^34^ utilizing the Fiji plug-in Color Inspector 3D^35^. This enables us to parameterize the color of each pixel in digitized images as a function of hue value (H) in an angular dimension ranging from 0° to 360°, while the saturation (S) and brightness (B) can be parameterized in linear dimension between 0 and 1. The hematoxylin staining is characterized as a “blue” background at H ≈ 250°, while the originally “red” PAS staining appeared as a “purple” subpeak at H ≈ 270°. Since the obtained histograms could be well fitted with two exponentially modified Gaussian distribution functions using IGOR software (WaveMetrics, Inc., OR, USA), the ratio of the PAS peak’s area and the total area under the curve was used as a precise index to quantitatively compare the glycogen content. As shown in Figs. 1A, B, the positions of peaks exhibited slight but non-negligible variations from sample to sample, explaining why the color deconvolution with defined color vectors failed.

### Activities of representative glycolytic enzymes

Based on the observation of a significant age-associated increase in abundance of proteins involved in the upper part of glycolysis, we have performed enzymatic assays of representative glycolytic enzymes in the CD34+ cells derived from different age groups. The methods have been described in a previous publication^36^. The activities of the following key enzymes for the glycolytic pathway, hexokinase, aldolase, triosephosphate isomerase, as well as adenylate kinase, were assessed. CD34+ cells were incubated on ice in ETC buffer (20 mM Tris–HCl pH 7.4, 250 mM sucrose, 50 mM KCl, 5 mM MgCl2) with 0.015% digitonin for 30 min. All enzymes added to the buffer for coupled enzymatic assays were purchased from Sigma-Aldrich and prepared from rabbit muscle. In all assay systems, activity was detected with and without addition of substrate to subtract unspecific background. *Hexokinase activity* was assayed as NADP reduction in ETC buffer containing 1 mM ATP, 1 mM glucose, 0.5 mM NADP, 0.05 U/ml glucose 6-phosphate dehydrogenase. *Trioso-phosphate isomerase* activity was assayed as NADH oxidation in ETC buffer containing 0.2 mM NADH, 4.9 mM DL-glyceraldehyde 3-phosphate, 0.4 U/ml α-glycerophosphate dehydrogenase (GPD1). *Pyruvate kinase activity* was detected as NADH oxidation in ETC buffer containing 0.1 mM phosphoenolpyruvate, 1 mM ADP, 0.5 mM NADH, 2 U/ml LDH. *Adenylate kinase activity* was assayed as NADP reduction in ETC buffer containing 1 mM ADP, 1 mM glucose, 0.5 mM NADP, 1 U/mL HK and 0.05 U/ml glucose 6-phosphate dehydrogenase at pH 7.5 and 37 °C.

A major problem with enzyme assays for human samples is biological variance, which cannot be accounted for by protein normalization or cell number count, thus rendering the data very noisy. To this end, we have normalized enzymes that have been associated to a highly glycolytic phenotype to the ones associated with a normal glycolytic state, the latter being pyruvate kinase “high affinity”. This internal normalization, literally within the glycolytic pathway itself, removes a lot of noise and accounts for individual differences of overall glycolysis activity.

### Classification of HPCs subsets by glucose uptake capabilities

The glucose uptake of the CD34+ cells was determined by using the Cayman’s Glucose Uptake Assay-Kit (Cayman Chemical, Michigan, USA). This kit detects directly glucose taken up in cells employing the fluorescently-labeled deoxyglucose analog 2-NBDG (2-[(7-nitro-2,1,3-benzoxadiazol-4-yl)amino]-d-glucose). An inhibitor of glucose transport mediated by GLUT1, Apigenin, a flavonoid, is used as a control. The advantage is that with this method, the cells remain vital after assessment and CD34+ cells could then be separated by a FACS-Sorter according to their levels of glucose metabolism^37^.

### Single-Cell RNA sequencing and data processing

In this study, we have re-analyzed the previously published scRNA seq datasets^10^. The details of the single cell RNA sequencing have been described in detail. Briefly, sequencing libraries from 192 single CD34+ + cells per donor were generated based on the smart-seq2 protocol of Picelli et al.^38^ and the tagmentation procedure of Hennig et al.^39^. Single CD34-positive cells were FACS sorted directly into 96-well plates containing 4.4 μl of lysis buffer per well. The lysates were incubated for 3 min at 72 °C and kept on ice while adding reverse transcription (RT) mix. Twenty-two cycles were applied for the PCR. Then, 25 μl nuclease-free water and 30 μl of SPRIselect (Beckman Coulter) (1:0.6 ratio) were added. After incubation, removal of supernatant, and drying, 13 μl nuclease-free water was applied for elution and 11 μl was taken for a second purification step. 40 μl nuclease-free water and 25 μl of SPRIselect (1:0.5 ratio) were added and after incubation, removal of supernatant, and drying, 13 μl nuclease-free water was applied for elution. 1.25 μl of the supernatant was used for tagmentation^39^. Tn5 was mixed with equal amounts of Tn5ME-A/Tn5MErev and Tn5ME-B/Tn5MErev and incubated at 23 °C for 30 min. Loaded Tn5 and sample were incubated for 55 °C for 3 min in 10 mM Tris–HCl pH 7.5, 10 mM MgCl2, and 25% dimethylformamide. The reaction was stopped with 0.2% SDS for 5 min at room temperature. The single-cell data preprocessing was performed using the programming language R. Raw reads were processed using the recent version of the Salmon pipeline (v0.9.1)^40^, with the index derived from transcriptome data from the hg38 build for mapping purposes (https://ftp.ensembl.org/pub/release-87/fasta/homo_sapiens/cdna/Homo_sapiens.GRCh38.cdna.all.fa.gz). The count matrix generated for individual transcripts across cells in each sample was then subjected to further processing using the Bioconductor package tximport^41^. The resulting count tables were analyzed using the Bioconductor package simpleSingleCell (version 1.2.0)^42^. The Work Flow of the computational processing of these single cell transcriptomics data is summarized in Figure S3 in the Supplementary Material. In Supplmentary Document, the Excel Tables summarizes the results of the Gene Ontogeny Analysis as well as the Pathway Analysis of the single-cell RNA sequencing data. For the GO analysis the R packages clusterProfiler^14^ and topGO^15^ were applied; for the pathway analysis we conducted a Fisher Exact test on pathways extracted from the Reactome Database^16^.

## Supplementary information

Supplementary Information 1.

Supplementary Information 2.
