# Supplementary material for: Glycogen accumulation, central carbon metabolism, and aging of hematopoietic stem and progenitor cells
Source: Sci Rep. 2020 Jul 14;10:11597. doi: 10.1038/s41598-020-68396-2 (PMC7360735; doi:10.1038/s41598-020-68396-2)
Supplement: Supplementary file 1 — Supplementary Information 1. [file 41598_2020_68396_MOESM1_ESM.pdf]

Supplementary Information to

Glycogen Accumulation, Central Carbon Metabolism, and Aging of Hematopoietic Stem and Progenitor Cells

Laura Poisa-Beiro[#1,2], Judith Thoma[#3,4], Jonathan Landry[5], Sven Sauer[6], Akihisa Yamamoto[4], Volker Eckstein[1], Natalie Romanov[7], Simon Raffel[1], Georg F. Hoffmann[6], Peer Bork,[2,7] Vladimir Benes[5], Anne-Claude Gavin[2,7,8], Motomu Tanaka*[3,4], Anthony D. Ho*[1,2,4]

1. Department of Medicine V, Heidelberg University, Im Neuenheimer Feld 410, 69120 Heidelberg, Germany
2. Molecular Medicine Partnership Unit Heidelberg, EMBL and Heidelberg University, 69120 Heidelberg, Germany
3. Physical Chemistry of Biosystems, Institute of Physical Chemistry, Heidelberg University, Im Neuenheimer Feld 253, 69120 Heidelberg, Germany
4. Center for Integrative Medicine and Physics, Institute for Advanced Study, Kyoto University, 606-8501 Kyoto, Japan
5. European Molecular Biology Laboratory (EMBL), Genomics Core Facility, Meyerhofstrasse 1, 69117 Heidelberg, Germany
6. Division of Child Neurology and Metabolic Diseases, Centre for Child and Adolescent Medicine, University Hospital Heidelberg, Im Neuenheimer Feld 430, 69120, Heidelberg, Germany
7. European Molecular Biology Laboratory (EMBL), Structural and Computational Biology Unit, Meyerhofstrasse 1, 69117 Heidelberg, Germany
8. Department for Cell Physiology and Metabolism, Centre Medical Universitaire, University of Geneva, Rue Michel-Servet 1, CH-1211 Geneva 4, Switzerland

# Equal contributions

* Corresponding authors:

anthony_dick.ho@urz.uni-heidelberg.de, tanaka@uni-heidelberg.de

Supplementary Tables:

Table S1

Activity in mOD/min per million cells

ID	Age	Gender	Aldolase	Adenylate kinase	Triose-phosphate isomerase	Hexo-kinase	Pyruvte Kinase High Affinity
214	29	m	9.54	5.47	100.50	4.02	20.52
219	19	f	15.08	5.96	105.75	4.13	21.72
221	71	f	2.12	3.32	71.60	1.27	5.81
309	21	f	0.87	3.61	34.70	0.29	7.03
308	23	m	6.38	10.95	125.17	1.14	18.54
307	44	m	10.74	17.07	172.77	2.50	22.43
305	62	f	7.36	11.27	163.33	2.29	11.28
303	69	m	7.78	11.58	109.26	1.66	9.58
302	59	m	7.64	10.85	176.54	2.09	6.23
214	29	m	0.46475	0.26667	4.89819	0.19573	
219	19	f	0.69446	0.27455	4.86869	0.19015	
221	71	f	0.36560	0.57155	12.32806	0.21785	
309	21	f	0.12355	0.51296	4.93634	0.04163	
308	23	m	0.34411	0.59049	6.75040	0.06167	
307	44	m	0.47883	0.76126	7.70273	0.11164	
305	62	f	0.65265	0.99898	14.47719	0.20257	
303	69	m	0.81267	1.20953	11.41047	0.17354	
302	59	m	1.22589	1.74099	28.33199	0.33516	
		Mean	0.57	0.77	10.63	0.17	
		SD	0.32	0.48	7.53	0.09	
z-Standard-ization	29	m	-0.339	-1.053	-0.762	0.289	
	19	f	0.377	-1.037	-0.766	0.227	
	71	f	-0.649	-0.415	0.225	0.538	
	21	f	-1.403	-0.537	-0.757	-1.443	
	23	m	-0.716	-0.375	-0.516	-1.218	

44	m	-0.296	-0.018	-0.389	-0.656
62	f	0.246	0.480	0.510	0.366
69	m	0.745	0.921	0.103	0.040
59	m	2.034	2.034	2.350	1.856

Table S1. Enzmye activities of representative enzymes for the glycolytic pathway

The activities of key enzymes for the glycolytic pathway, aldolase, hexokinase, triosephosphate isomerase, as well as adenylate kinase, were assessed in 9 human subjects (age range: 19 years to 71 years). A major problem with enzyme assays for human samples is biological variance. To resolve this challenge. we have normalized enzymes that have been associated to a highly glycolytic phenotype to the ones associated with a normal glycolytic state. the latter being pyruvate kinase "high affinity". This internal normalization was able to eliminate a lot of noise and accounts for individual differences of overall glycolysis activity.

Table S2

		Aldolase	Adenylate-kinase	Triose-phosphate isomerase	Hexokinase
	19	0.377	-1.037	-0.766	0.227
	21	-1.403	-0.537	-0.757	-1.443
	23	-0.716	-0.375	-0.516	-1.218
One-sided t-test	29	-0.339	-1.053	-0.762	0.289
	44	-0.296	-0.018	-0.389	-0.656
	59	2.034	2.034	2.350	1.856
	62	0.246	0.480	0.510	0.366
	69	0.745	0.921	0.103	0.040
	71	-0.649	-0.415	0.225	0.538
	p-value	0.07	0.02	0.01	0.04
Correlation coefficient trend	R^2	0.21	0.43	0.42	0.33

Mean	Aldolase	Adenylate-kinase	Triose-phosphate isomerase	Hexokinase
Young	-0.52	-0.75	-0.70	-0.54
Old	0.59	0.75	0.80	0.70

Table S2. Statistical analysis

Statistical analysis of the changes in enzyme activities of the glycolytic pathway, aldolase, hexokinase, triosephosphate isomerase, as well as adenylate kinase. After z-standardization. one-sided t-test was used based on the hypothesis that activities were higher in older subjects than younger subjects. Subject at age 44 was excluded from the analysis as this represents the median.

Table S3

Myeloid	Lymphoid
PRKG2	IRF8
GUCY1A3	ADA
GUCY1B3	PAG1
TBXAS1	MZB1
PTGS1	DNTT
PML	LAT2
PSTPIP2	RCSD1
TFRC	CD19
	IL7R
	EBF1
	MME
	PAX5

Table S3: Gene markers for myeloid- versus lymphoid differentiation potentials

List of gene markers for the single cell RNA analysis that are considered to be specific for myeloid or lymphoid differentiation.

Table S4:

Gene Name	Description
PYGB	Glycogen phosphorylase that regulates glycogen mobilization (PubMed:27402852). Phosphorylase is an important allosteric enzyme in carbohydrate metabolism (PubMed:3346228). Enzymes from different sources differ in their regulatory mechanisms and in their natural substrates (PubMed:3346228). However, all known phosphorylases share catalytic and structural properties (PubMed:3346228).
PYGL	Phosphorylase is an important allosteric enzyme in carbohydrate metabolism. Enzymes from different sources differ in their regulatory mechanisms and in their natural substrates. However, all known phosphorylases share catalytic and structural properties.
AGL	Multifunctional enzyme acting as 1,4-alpha-D-glucan:1,4-alpha-D-glucan 4-alpha-D-glycosyltransferase and amylo-1,6-glucosidase in glycogen degradation.
HK1	Glycerol-3-phosphate dehydrogenase, mitochondrial: This subpathway is part of the pathway glycerol degradation via glycerol kinase pathway, which is itself part of Polyol metabolism.
PFKM	Glycerol-3-phosphate dehydrogenase, mitochondrial: This subpathway is part of the pathway glycerol degradation via glycerol kinase pathway, which is itself part of Polyol metabolism.
TALDO1	Glycerol-3-phosphate dehydrogenase, mitochondrial: This subpathway is part of the pathway glycerol degradation via glycerol kinase pathway, which is itself part of Polyol metabolism.
ALDOC	Glycerol-3-phosphate dehydrogenase, mitochondrial: This subpathway is part of the pathway glycerol degradation via glycerol kinase pathway, which is itself part of Polyol metabolism.
TPI1	Glycerol-3-phosphate dehydrogenase, mitochondrial: This subpathway is part of the pathway glycerol degradation via glycerol kinase pathway, which is itself part of Polyol metabolism.
GPD2	Glycerol-3-phosphate dehydrogenase, mitochondrial: This subpathway is part of the pathway glycerol degradation via glycerol kinase pathway, which is itself part of Polyol metabolism.

Table S4: List of glycolytic proteins used in scRNA seq analysis.

Supplementary Figures:

Figure S1:

Color Vector	Channel 0<br>RGB image	Channel 1<br>hematoxylin	Channel 2<br>PAS or eosin	Channel 3<br>= Channel 0 – (Channel 1 + Channel 2)
(A) Hematoxylin-PAS	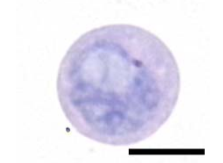	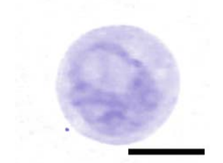	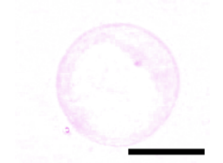	
(B) Hematoxylin-Eosin	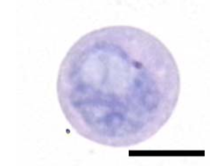	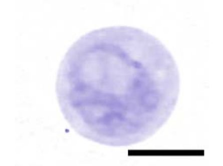		

Figure S1: Semi-quantitative Assays of PAS+ content

Example of the commonly used color deconvolution in RGB performed with color vectors for HPC from healthy old donor stained by (A) hematoxylin-PAS and (B) hematoxylin-eosin. Channel 0 is the RGB image after correcting the background. The subtraction of Channel 1 (hematoxylin) and Channel 2 (PAS or eosin) from Channel 0 should result in no signal in Channel 3. The remaining signals in Channel 3 clearly indicate that the color deconvolution failed. Scale bar: 10 µm. Software: Fiji plug-in Color Inspector 3D.

Figure S2:

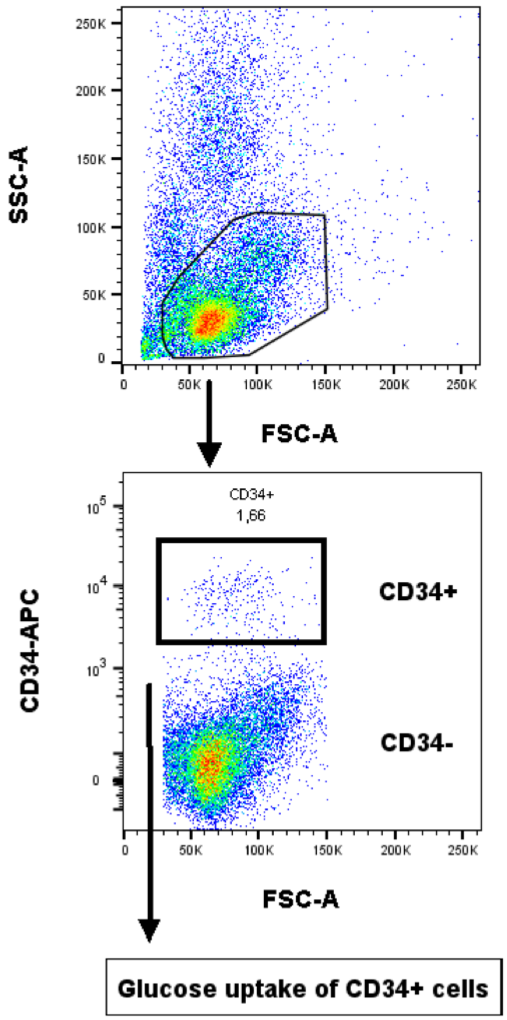

Figure S2: Example of the FACS gating strategy for CD34+ cells

Cells are sorted based on their CD34 expression. Software: FlowJo v10.6.2.

Figure S3:

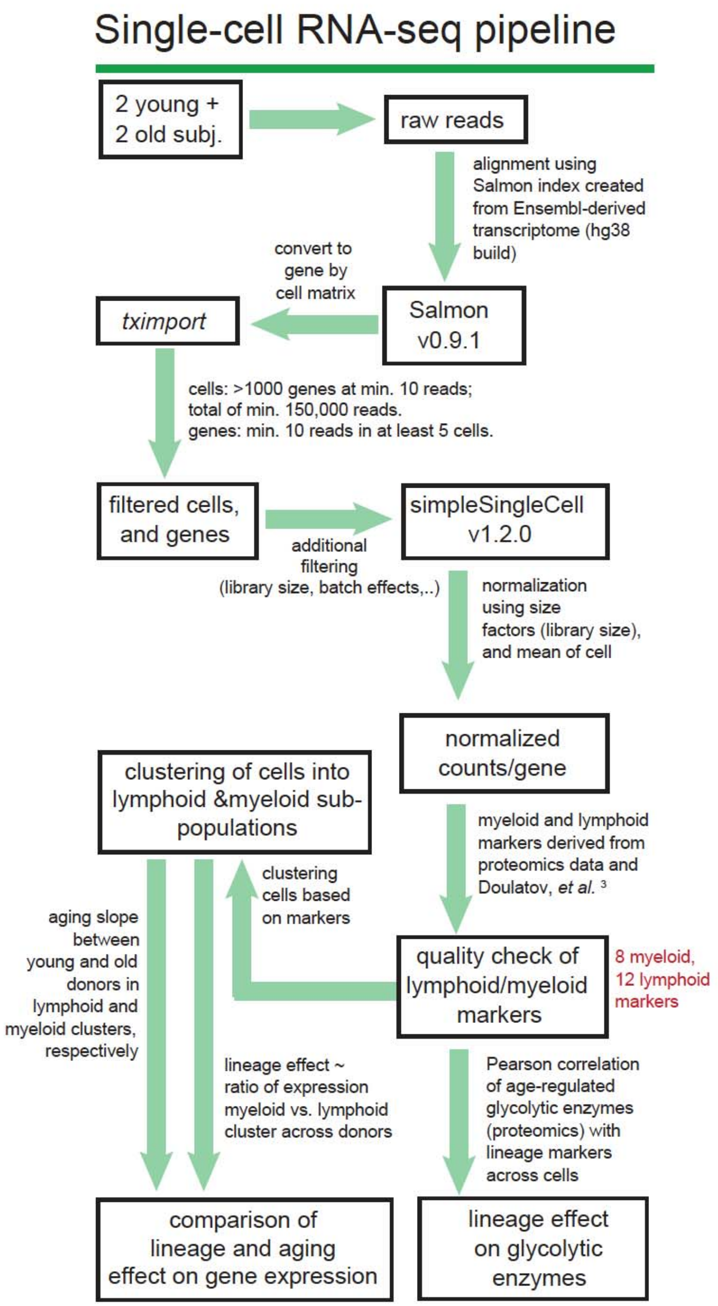

Figure S3: Single-cell RNA-seq processing pipeline after acquisition of raw reads.

Access number for the scRNA seq data:
https://www.ncbi.nlm.nih.gov/geo/query/acc.cgi?acc=GSE115353

Figure S4

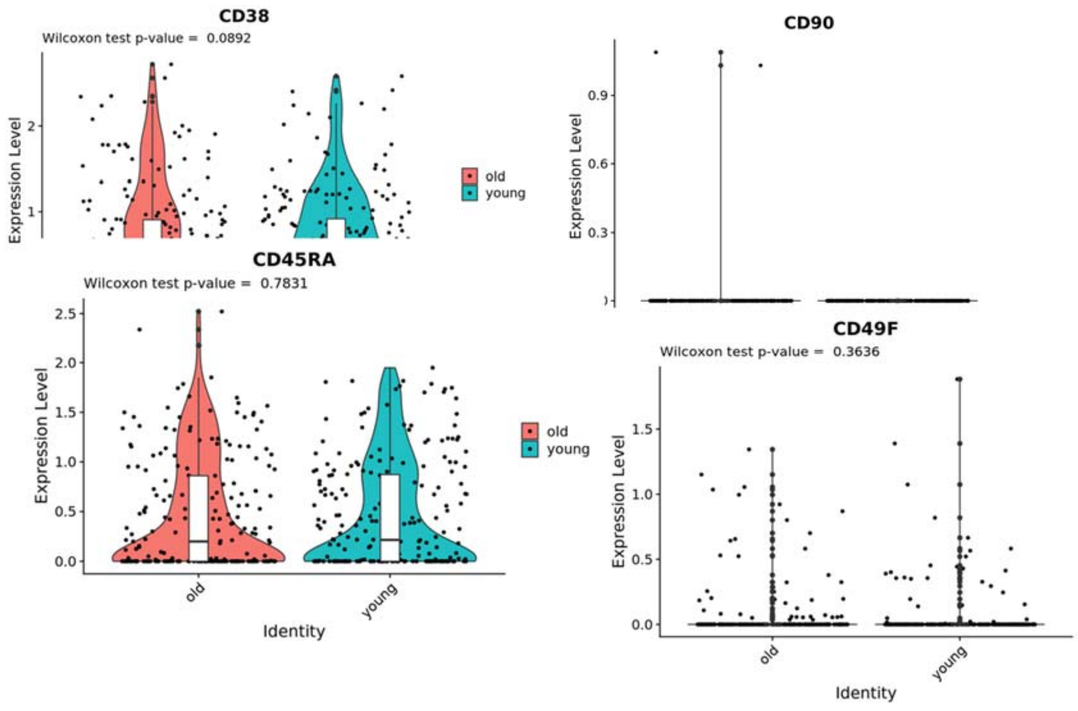

Figure S4: Aging and Surface Markers for HPCs.

Relationship between aging of CD34+ cells and co-expressions of CD38, CD90, CD45RA and CD49f, based on single-cell transcriptomics study. Software: Python 2.7
